# YAP1 Is a Potential Predictive Molecular Biomarker for Response to SMO Inhibitor in Medulloblastoma Cells

**DOI:** 10.3390/cancers13246249

**Published:** 2021-12-13

**Authors:** Gustavo Alencastro Veiga Cruzeiro, Taciani de Almeida Magalhães, Graziella Ribeiro de Sousa, Ricardo Bonfim Silva, Carlos Alberto Oliveira de Biagi Junior, Pablo Ferreira das Chagas, Rosane Gomes de Paula Queiroz, Carlos Alberto Scrideli, Luiz Gonzaga Tone, Elvis Terci Valera

**Affiliations:** 1 Department of Pediatrics, Ribeirão Preto Medical School, University of São Paulo, Hospital das Clínicas Ribeirão Preto, Av. Bandeirantes 3900, Ribeirão Preto 05468901, São Paulo, Brazil; rosane@fmrp.usp.br (R.G.d.P.Q.); scrideli@fmrp.usp.br (C.A.S.); lgtone@fmrp.usp.br (L.G.T.); etvalera@hcrp.usp.br (E.T.V.); 2 Department of Pediatric Oncology, Dana-Farber Boston Children’s Cancer and Blood Disorders Center, Harvard Medical School, 450 Brookline Avenue, Boston, MA 02215, USA; 3 Broad Institute of Harvard and MIT, Cambridge, MA 02142, USA; 4 Department of Genetics, Ribeirão Preto Medical School, University of São Paulo, Av. Bandeirantes 3900, Ribeirão Preto 05468901, São Paulo, Brazil; taciani_magalhaes@hms.harvard.edu (T.d.A.M.); graziellasousa@usp.br (G.R.d.S.); bonfim.ricardo@usp.br (R.B.S.); biagi@usp.br (C.A.O.d.B.J.); pablochagas@usp.br (P.F.d.C.); 5 Department of Cell Biology, Harvard Medical School, 240 Longwood Avenue, Boston, MA 02115, USA

**Keywords:** SHH medulloblastoma, YAP1, SMO inhibitor

## Abstract

**Simple Summary:**

Medulloblastoma (MB) is the most common malignant brain tumor in childhood. Currently, MB is assigned in four molecular subgroups (SHH, WNT, Group 3, and Group 4) and subtyped in 12 variants. The alpha subtype of the SHH subgroup bears *TP53* mutation and is considered very high risk by the World Health Organization (WHO). In the current study, we have investigated the role of YAP1 expression in SHH MBs. Herein, we show: (1) SHH MB patients genotypically profiled as resistant to SMOi and the aggressive alpha subtype overexpress YAP1; (2) SHH-like cell lines bearing *TP53* mutation show improved responsiveness to SMOi upon YAP1 depletion; (3) Sonidegib (smoothened inhibitor) and Verteporfin (YAP1 inhibitor) synergize at specific doses; (4) distinct cell populations in the single-cell RNA-seq patient setting express YAP1 and *SMO.*

**Abstract:**

Advances in genomics have led to the identification of twelve relevant molecular subtypes within medulloblastoma (MB). The alpha subtype of Sonic hedgehog-driven MB is resistant to therapy (including smoothened inhibitors) due to activation of genes from the non-canonical SHH pathway, such as *MYCN*, *YAP1*, or *TP53*. Using retrospective cohort microarray data, we found that *YAP1* is overexpressed in SHH alpha MB and patients profiled as resistant to SMO inhibitors compared to good responders. Here, we performed *YAP1* depletion via CRISPR/Cas9 in two in vitro models of SHH-like MB cells and found that this protein is involved in responsiveness to the SMO inhibitor regarding proliferation, apoptosis, and colony formation. Further, considering the synergic combination of YAP1 depletion with SMO inhibition, we assessed single-cell RNA-seq data from five patients and found that *SMO* and *YAP1* are enriched within cells of SHH MB. Importantly, our data suggest that *YAP1* is not only a reliable biomarker for cellular response to SMOi but may indicate prospective testing of combination therapy using YAP1 and SMO inhibitors in preclinical models of SHH MB.

## 1. Introduction

### The Context of YAP1 in SHH Medulloblastoma

Medulloblastoma (MB) is a heterogeneous malignant brain tumor that commonly affects infants and children. Current consensus assigns MB in 4 subgroups (WNT, SHH, Group 3, and Group 4) with subclassifications into 12 distinct subtypes [1]. Notably, these subtypes bear distinct prognoses and molecular features [2]. SHH medulloblastoma (SHH MB) is divided into four subtypes corresponding to alpha, beta, gamma, and delta. [1,2]. The SHH alpha subtype shows the *TP53* mutation (considered a very high-risk factor by WHO 2016 and 2021) and *MYCN/GLI2* amplification. The SHH beta subtype occurs in infants and often shows metastasis (poor outcome). The SHH gamma subtype also occurs in infants and shows an extensive nodularity histology and is associated with a better outcome. Finally, the SHH delta subtype shows mutations in *TERT* promoters and occurs mostly in adults. In summary, the SHH alpha subtype and beta subtype are the most aggressive and are categorized as very high-risk. SHH alpha- and beta-subtyped patients show unmet needs that often relapse or succumb to the disease. This poor scenario highlights the urgent need for novel therapeutic targets and predictive biomarkers. Likewise, one of the leading causes of death in children is tumor recurrence, led by therapy resistance due to remaining specific subpopulations unaffected by treatment [3]. In the context of SHH MB, resistance to targeted therapy using agents such as SMO inhibitors (e.g., Sonidegib and Vismodegib) is frequently associated with alterations in genome and transcriptome, such as *TP53* mutation and transcriptional activation of oncogenes such as *MYCN* and *YAP1* [1,4,5]. Overexpression of *YAP1* contributes to aggressiveness in several tumor types by inducing proliferation, aggressiveness, tumor initiation, and stemness. In other cancers, it is a biomarker of good prognosis. Notably, the ambiguous feature highlights YAP1 as a promiscuous transcriptional co-activator that binds to partners to exert distinct functions depending on the downstream effectors of signaling pathways. Recently, we revealed that YAP1 restricts cellular migration through *CTGF* expression and promotes cellular proliferation in SHH-like MB cell lines [6]. In addition, other authors showed that YAP1 is induced by smoothened, a G-protein coupled receptor and transmembrane protein of the Sonic hedgehog pathway (SHH). Nonetheless, it is still unclear whether it is a single-axis canonical activation or if YAP1 is induced by non-canonical effectors downstream of SMO [7]. Furthermore, although this intrinsic relation of YAP1 with Shh is known, the YAP1 function in SHH MB biology remains unclear in the clinical context [7]. Since SHH MB patients relapse more than metastasize, we sought to investigate if *YAP1* plays a role in targeted therapy resistance, specifically to the SMO inhibitor, Sonidegib. CRISPR-based knockout and single-cell cloning are increasingly applied to targeted drug combinations investigations in order to lead to synthetic lethality [8]. Herein, we have assessed transcriptomic public records of two microarray datasets and found that YAP1 is explicitly overexpressed in patients profiled as poor responders to SMO inhibitors (e.g., Sonidegib, Vismodegib) and in SHH medulloblastoma subtype alpha. Using two in vitro models of SHH-like MBs, we performed CRISPR/Cas9 knockout and single-cell cloning. We found that YAP1 dramatically reduces the cellular response to Sonidegib (SMOi) regarding proliferation, apoptosis, and colony formation. Lastly, we have withdrawn single-cell data from public data sets explored in recent studies and found that *SMO* and *YAP1* are enriched in SHH MB cells. These findings open new avenues for synergic combination therapy utilizing a YAP1 inhibitor in association with an SMO inhibitor.

## 2. Methods

### 2.1. Cell Culture

The human MB cell line DAOY and UW228 (SHH-like subgroup; *TP53* mutated—c.725G > T and SHH-like subgroup, c.464C > A, respectively) [9] was cultured using RPMI-1640 medium supplemented with 10% fetal bovine serum (Gibco BRL, Carlsbad, CA, USA), penicillin (100 U/mL) and streptomycin (100 lg/mL) in a humidified atmosphere with 5% CO_2_ at 37 °C. Cell line authentication by short tandem repeats (STR) DNA profiling analysis was performed to assure authenticity and avoid cellular cross-contamination. Since DAOY and UW228 are commercially available cell lines and not primary cultures, SHH-like subgroup cell lines are best assigned as SHH subgroups according to comparative transcriptional profiles when compared to medulloblastoma patients [9,10,11].

### 2.2. Generation of CRISPR/Cas9 Single-Clones YAP1 Depleted Cells

Briefly, guide RNA (crRNA) utilized to target YAP1 (TGCCTCAGACCGTGCCCATG) was designed by the CHOP_CHOP tool that broadens regions with 100% homology of Exon 2 isoforms. Cell lines expressing Cas9 DAOY and UW228 were previously transduced and selected with a pLenti_Cas9_puro vector. After establishing cell lines that constitutively express Cas9, crRNA for YAP1 + tracrRNA was transfected using the Dharmafect reagent. Following the manufacturer’s recommendations, after 48 h, transfected cells were submitted to limited dilution for single-cell cloning [12], being 50 cells per 96-well plate. Twenty Clones were picked, expanded isolated, and progressively passaged from a 96-well plate, 24-well plate, and T75 flask. Protein depletion was assessed via immunoblotting to confirm YAP1 knockout efficiency. As negative controls, we transfected cells with a non-targeting control (non-targeting guide crRNA + tracRNA). After expansion, cells were lysed and derived-clones protein lysates were assessed via immunoblotting as described below.

### 2.3. Immunoblotting

Cells were lysed in RIPA buffer on ice for 15 min. Lysates were probed with the primary antibodies anti-YAP1 (1:1000 Cell Signaling #14074, Danvers, MA, USA) and GAPDH (1:1000 Cell Signaling #14C10). Respectively, secondary antibodies were used: 1:2500 anti-mouse horseradish peroxidase (HRP)-linked and anti-rabbit HRP-linked (1:2000; #7076; #7074; Cell Signaling). Detailed methods and antibody optimization can be found in our previous article [6].

### 2.4. CCK8 Proliferation Assay and Doubling Time

A cell proliferation assay was performed with a sensitive colorimetric assay—Cell Counting Kit-8 (CCK-8)—and the protocol was applied according to the manufacturer’s protocol (Dojindo Laboratories, Kumamoto, Japan). The full protocol can be accessed from our previous publication [6].

### 2.5. Retrospective Analysis of Microarray Data

*YAP1* expression in MB subtypes was assessed in two datasets: GSE85217 for medulloblastoma subtypes, and GSE49243 for patients profiled as responders and non-responders [4]. Briefly, according to Kool and colleagues, within the SHH molecular subgroup, there are non-responsive patients to SMO inhibitors (characterized by genomic alterations such as mutations in *TP53* or *MYCN*). On the other hand, there is another subset of patients with a relatively more stable genome that responds to SMOi (e.g., *TP53* WT patients). This prediction has been tested in SHH PDX models [4] and to a lesser extent, in patients (NCT01708174). In summary, the categorization of non-responders and responders was created based on a genomic profile. Other in silico analyses were performed through the R2 Genomic Analysis and Visualization platform (http://r2.amc.nl, accessed on 10 October 2021). Expression values were normalized via the MAS5.0 Affymetrix algorithm for producing gene expression signals. Reads were scaled using log2 or Z-score.

### 2.6. Colony Formation Assay

Cells were plated at a concentration of 500 cells per well in a 6-well plate and were treated according to the IC50 previously established (Figure S1a). After treatment, cells were recovered using fresh media for 15 days. On day 15, cells were fixed with methanol and stained using GIEMSA. A detailed protocol can be found in studies previously published by our group [13,14].

### 2.7. Annexin V/Propidium Iodide Staining to Detect Apoptotic and Necrotic Cells

After treatment at established doses and time points (Figure S1a), apoptotic and necrotic cells were identified via flow cytometry using BD FACScalibur (Becton, Dickinson and Company, Franklin Lakes, NJ, USA). Annexin-V and PI were utilized to detect apoptotic and necrotic cells, respectively. Experiments were performed thrice.

### 2.8. Statistical Analysis

Data were presented as the mean ± SD. Statistical analyses were performed using one-way analysis of variance (ANOVA) followed by Bonferroni’s multiple comparisons test. A two-way ANOVA followed by Dunnett’s test were performed to analyze the combined effect of Verteporfin with Sonidegib. Synergism analysis was defined by the Chou Talalay method using Calcsyn software (Version 2.0, Biosoft, Cambridge, UK) (C > 1.0 is synergic) [15]. A significant effect or statistically relevant result were defined by the *p*-values [* *p* < 0.05, ** *p* < 0.01, *** *p* < 0.001].

### 2.9. Single-Cell RNA-Seq Analysis

The scRNA-seq data from medulloblastoma were acquired from the Gene Expression Omnibus (GEO) database under the series number GSE119926, which contains data of five SHH MB samples generated using the Illumina NextSeq 500 system. The filtered feature-barcode matrix was used in the following analysis. All additional analyses were performed using Seurat v3. Cells with less than 200 and greater than 3000 genes detected were excluded from the analysis. For clustering of all cells, we assigned scores for S and G2/M cell cycle phases based on previously defined gene sets using the CellCycleScoring function. The data were normalized using the NormalizeData function and the identification of features that were outliers was performed using the FindVariableFeatures function, both with default parameters. The ScaleData function was used to scale and center features in data regressing out against the number of UMIs per cell, S phase score, and G2/M phase score. Scaled data were used as an input into PCA based on variable genes. The first 25 PCs were used to generate the UMAP projection. All information about the patient’s subgroup, sample setting, and cellular population can be found in Figure S1c [16,17,18].

## 3. Results

*YAP1* is overexpressed in predicted non-responsive SHH MB patients and SHH alpha subtype patients.

**Figure 1:**
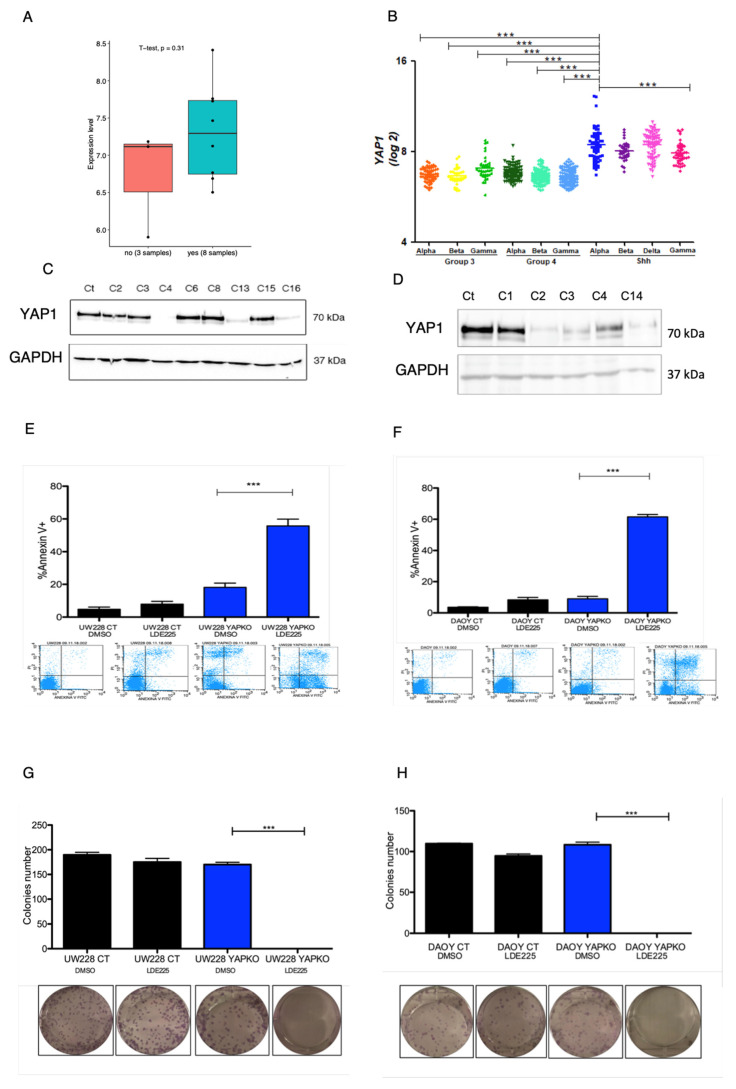
(**A**) *YAP1* is overexpressed in patients resistant to SMOi (blue bar) compared to good SMOi responders (red bar) (*p* < 0.05). (**B**) Microarray in silico analysis of 693 MB primary samples shows that *YAP1* is overexpressed in SHH MB compared to Group 3 and Group 4, and higher in SHH-α subtype. (**C**) Cropped image blot of UW228 CRISPR/Cas9 single-cell clones (C’s) with depletion of YAP1 protein at 70 kDa and expression of endogenous GADH (37 kDa) (complete image can be found in Supplementary Information). (**D**) Cropped image blot of DAOY CRISPR/Cas9 single-cell clones (C’s) with depletion of YAP1 protein at 70 kDa and expression of endogenous GAPDH (37 kDa) (complete image can be found in Supplementary Information). (**E**) DAOY YAP1 knockout cells (clone C2) (blue bar) under SMOi had high increase in apoptosis (Annexin V+) compared to control cells (black bar), and untreated control. (**F**) UW228 YAP1 knockout cells (clone C4) (blue bar) under SMOi had high increase in apoptosis (Annexin V+) compared to control cells (black bar) and untreated control. (**G**) UW228 YAP1 knockout cells (blue bar) under SMOI did not form colonies compared to control cells (black bar) and untreated control. (**H**) DAOY YAP1 knockout cells (blue bar) under SMOi did not form colonies compared to control cells (black bar) and untreated control. A significant effect or statistically relevant result were defined by the *p*-values (*** *p* < 0.001).

We first aimed to assess *YAP1* expression in SHH MB patients. Therefore, we utilized the Gene Expression Omnibus GSE49243 and GSE85217 microarray datasets containing transcription data of 11 patients profiled as responsive and non-responsive to SMO inhibitor and 693 patients assigned according to MB subtypes. Besides the limited number of patients in GSE49243 and the *t*-test showing no significance, *YAP1* is overexpressed in patients characterized by *TP53* mutation and other genes promoters of genomic instability. These patients were profiled as non-responsive to SMO inhibitor [8] and compared to responsive to SMO (patients without these key alterations) [3] (*p* = 0.31) (Figure 1A). In addition, expression analysis on the GSE85217 dataset (693 patient cohort) showed that *YAP1* is overexpressed specifically in the aggressive subtype alpha (Figure 1B), compared to other subgroups and subtypes of medulloblastoma.

### 3.1. YAP1 Knockout in MB Cell Lines Bearing TP53 Mutation Leads to Anti-Proliferative and Pro-Apoptotic Response to Sonidegib

**Figure 2:**
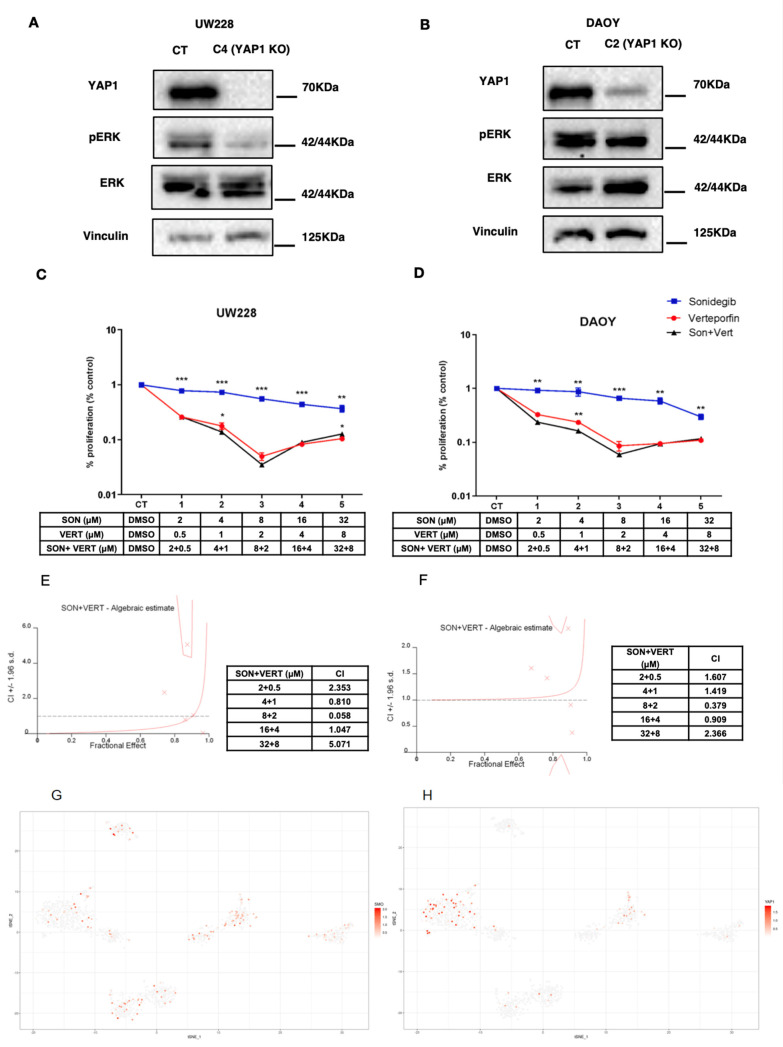
(**A**) UW228 control versus single-cell clone C4 YAP1 KO. YAP1-depleted cells show reduced levels of pERK at Serine 42/44. (**B**) DAOY Control versus single-cell clone C2 YAP1 KO—YAP1-depleted cells showing reduced levels of pERK at Serine 44. (**C**) UW228 combination of Sonidegib with Verteporfin (black line) decreases proliferation compared to Sonidegib (blue line) only, and to a lesser extent, Verteporfin (red line) only. Drug concentrations scheme are shown below. (**D**) DAOY combination of Sonidegib with Verteporfin (black line) decreases proliferation compared to Sonidegib (blue line) only, and to a lesser extent, Verteporfin (red line) only. Drug concentrations scheme are shown below. (**E**) UW228 combination index values estimated by Calcsyn using the Chou–Talalay method (CI > 1.0 are considered synergic). (**F**) DAOY combination index values estimated by Calcsyn using the Chou–Talalay method (CI > 1.0 are considered synergic). (**G**) *SMO* expression within single cells across SHH MB samples. (**H**) *YAP1* expression within single cells across SHH MB samples. A significant effect or statistically relevant result were defined by the *p*-values (* *p* < 0.05, ** *p* < 0.01, *** *p* < 0.001).

In order to investigate whether YAP1 expression weakens SMOi response, we depleted *YAP1* in two cell lines DAOY and UW228, authenticated by STR profile, that were previously assigned by our group as SHH-like with *TP53* mutation [9]. CRISPR/Cas9 knockout mediated by transfection successfully depleted the YAP1 protein (Figure 1C,D and Figure S1a–c) and single-cell clones were isolated in 96-well plates through the limited dilution method [12]. After harvesting YAP1 knockout clones, cells were expanded and proceeded for functional assays (proliferation, apoptosis, and colony formation). These assays were performed by incubating transfected CRISPR/Cas9 non-targeting control cell lines and *YAP1* CRISPR/Cas9-engineered knockout with SMOi on a previously established dose IC50 (*p* < 0.001) (Figure S2a). Interestingly, while YAP1-depleted cells showed a decrease in proliferation rate (*p* < 0.001) (Figure S2a) and a significant increase in apoptosis levels, (Figure 1E,F) they failed in forming viable colonies (Figure 1G,H) compared to the control (*p* < 0.001). Our data corroborate with previous studies that highlight YAP1 as a promoter of drug resistance through several pathways, including the RAF, MEK, ERK pathways [19]. Interestingly, we found that DAOY and UW228 YAP1-knockout/depleted cells showed a significant reduction in ERK phosphorylation at Serine 42 and 44 (Figure 2A,B). Additionally, using a bioinformatic tool composed from an algorithmic annotation of functional Roles for Components of 3044 Human Molecular pathways [10], we found pathways correlated with YAP1 expression in the SHH MB cohort of GSE85217 datasets. Out of 3044 pathways assessed, we found the top 5 correlated pathways with YAP1 expression in SHH medulloblastoma samples: (1) basal cell carcinoma, (2) hedgehog signaling, (3) ketogenesis, (4) SMAD2_SMAD3, inflammasomes, (5) PDGF signaling pathway. Interestingly, we found a duality in the Akt pathway. While Akt signaling pathway p73-mediated apoptosis is the most negatively correlated with YAP1 expression, the Akt signaling pathway cell cycle is positively correlated (R = 0.59, *p* = 2.84 × 10^−73^, Rank: 100). In addition, PDGF (R = 0.73, *p* = 3.90 × 10^−132^, Rank: 7) and mTOR pathway inflammation stress resistance (R = 0.63, *p*= 1.47 × 10^−87^, Rank: 60) (Table S1)

In the context of MB, *YAP1* seems to function restrictedly through a chromatin remodeler, such as HELLS [7] and promote radio resistance through the IGF pathway axis as a downstream target of the hedgehog pathway [20]. Since SMOi is a hedgehog blocker (through SMO inhibition), the combination with YAP1 depletion potentially impaired alternative therapeutic escape mechanisms that may be driven by *YAP1* expression. Indeed, using RNA-seq data from 46 SHH MB patients, we found that *YAP1* expression has a positive correlation with *GLI1* and *GLI2* (Figure S2b). Our findings uncover a potential combination of the YAP1 inhibitor with SMOi that might reveal a new SHH MB vulnerability.

### 3.2. Sonidegib and Verteporfin in Specific Concentrations Show a Synergic Effect in DAOY and UW228 Cell Lines

Next, we aimed to assess if the combination of Verteporfin (YAP1 inhibitor commonly used for macular degeneration) with Sonidegib in DAOY and UW228 may provide a synergic effect. Using the Chou–Talalay method [21], we found that Verteporfin and Sonidegib are synergic in doses that showed a combination index higher than 1, which is doses 1, 4 and 5 for UW228 and 1, 2, and 5 for DAOY (combination index—CI > 1.0) (Figure 2C–F)

### 3.3. Single-cell RNAseq Analysis Reveals That SMO, a Target of Sonidegib, and YAP1 Is Expressed in Distinct Subpopulations of MB Cells

Next, to assess the translational potential of prospective YAP1 inhibition combined with SMOi, using single-cell RNA-seq dataset GSE119926, we assessed the expression of *SMO* and *YAP1* in regard to subpopulations of cells within three SHH MB patients and two SHH patient-derived xenografts (PDX) [18] (Figure S2c). Interestingly, in two (SJ577 and SJ454) out of three SHH MB patient samples, *YAP1* and *SMO* were distinctively expressed across cell populations, while *YAP1* expression was absent in the PDX setting (RCMB18 and RCMB24) (Figure 2G,H). Remarkably, another study evaluating an SHH MB *Atoh1-Ptch1* mouse model utilized Verteporfin [22], a YAP1 inhibitor, and found that most cellular populations enriched with YAP1 are resistant OLIG2-positive cells [23].

## 4. Discussion

Increasing evidence shows that *YAP1* drives oncogenesis in several types of cancer; however, its role in MB remains elusive. Nonetheless, several authors described an intrinsic role of *YAP1* in the Sonic hedgehog pathway in MB, specifically in the SHH subtype. It is unclear whether this transcriptional co-activator induces SHH MB tumorigenesis and aggressiveness. Several authors described its role in the biology of granule cells (hypothetical cell of origin of SHH MB), specifically by promoting radioresistance. Authors have also described that *YAP1* amplification (not overexpression) is an important marker for prognosis and subtyping SHH MB [5]. In addition, the YAP1 protein is highly detected in the SHH subtype and shows a slight preference for medulloblastoma with extensive nodularity (MBEN) histological subtype (through immunohistochemistry).

Our study shows overexpression of *YAP1* in SHH MB patients assigned as alpha subtype, the most aggressive entity that bears the *TP53* mutation. Importantly, SHH MB patients with a mutation on the *TP53* gene are considered very high risk by the World Health Organization (WHO) and show no response to therapy, including chemoradiation and other small molecule inhibitors currently on trial. Intuitively, we tested Sonidegib, a potent blood–brain barrier-penetrating SMO inhibitor [15] (SHH pathway blocker), on two *TP53*-mutated cell lines with an SHH-like signature [8] and found poor responsiveness regarding cellular proliferation, apoptosis, and colony formation. Interestingly, YAP1 knockout mediated by CRISPR/Cas9 and single-cell cloning revealed that YAP1 is dispensable for cell survival. However, when exposing YAP1 knockout cells to Sonidegib, they dramatically lose viability and undergo apoptosis. This finding highlights a potential axis of synthetic lethality where SMO, an effector of the Sonic hedgehog pathway, is inhibited by Sonidegib, and *YAP1* depleted by CRISPR/Cas9, leading to a responsive phenotype. Importantly, it was possible to reasonably reproduce these findings in two *TP53* SHH-like cell lines. The interplay between the YAP1/Hippo pathway and *TP53* has become more relevant in recent years, given the role of P53 and its family members (P63, P73, and truncated P63) in impacting YAP1 subcellular localization [24]. The transcriptional regulatory network composed of these transcription factors can either induce YAP1 (through P63) or block its translocation to the nuclei (wildtype functional P53) [24]. Mutation in the DNA binding site of *TP53* likely leads to loss of its optimal conformation (as found in DAOY and UW228 cell lines), consequently impairing its function as a tumor suppressor gene. This phenomenon highly impacts YAP1 nuclear localization and activity as a transcriptional co-activator. however, little is known about YAP1 nuclear co-activator partner in SHH MB. It is also imperative to understand the cellular architecture and transcriptional programs within SHH MB as an entity. Assessing gene expression in distinct subcellular populations and evaluating their distribution may shed new light on the potential combination of small molecule inhibitors. Although our study preliminarily showed a synergic effect, the assessment of Verteporfin and Sonidegib in additional models, specifically, in neurosphere and primary SHH MB, may provide fundamental information about the benefit of using such a strategy. As shown by the pathway enrichment analysis, YAP1 is co-expressed and is potentially co-activated with the hedgehog pathway in SHH MB. Although the mechanism in which the hedgehog and YAP1 pathway co-opts remains elusive, we found a potential axis via Akt-MEK-ERK. In particular, MEK/ERK is required for hedgehog activation in many tissue types and enhances proliferation. In SHH MB, MEK/ERK may work as an independent axis but is important for boosting proliferation. This finding suggests that ERK and GLI2 (downstream effectors of YAP1 and SHH pathway in MB, respectively) may synergistically increase tissue malignancy and growth in SHH MB. Nonetheless, our study evaluated *YAP1* and *SMO* expression across cell populations and found their distinct expression within SHH MB patients and PDX samples. These findings point towards Sonidegib and Vertperforin, both BBB penetrants [15,23], as candidates for preclinical testing on SHH MB.

## 5. Conclusions

CRISPR screening and single-cell technologies are valuable tools for discovering novel targeted drug combinations that can shed light on synthetic lethality. In our context, we found that SMO inhibitor alone is not enough to block proliferation and kill cells. Similarly, when we depleted only YAP1, no effect was seen. However, the combination of YAP1 KO with SMOi dramatically reduced proliferation by causing cell death. Overall, our study found that: (1) *YAP1* is overexpressed in patients profiled as resistant to SMOi and in the aggressive alpha subtype, (2) *SHH-*like cell lines bearing *TP53* mutation showed improved responsiveness to SMOi upon YAP1 depletion, and (3) *YAP1* and *SMO* are expressed in distinct cell populations in a single-cell RNA-seq patient setting. Lastly, our study contributes to (1) identifying YAP1 as a novel potential predictive biomarker for SMO inhibitors and prospectively (2) adding information for further evaluations that aim to develop new molecularly targeted combinatorial therapies.

## Data Availability

The data presented in this study are available in this article (and supplementary material).

## Supplementary Materials

The following are available online at https://www.mdpi.com/article/10.3390/cancers13246249/s1, Figure S1: (a) Colorimetric acquisition using Bio-rad Chemidoc of the nitrocellulose membrane that contains a prestained ladder and proteins derived from UW288 single cell colonies. (b) Chemiluminescent acquisition of nitrocellulose membrane from Figure S2a incubated with rabbit monoclonal antibody YAP1 (CST-D8H1X) Cell Signaling Technologies and Secondary HRP-Rabbit (7074S). Figure S2c Chemiluminescent acquisition of nitrocelulose membrane from (c) after restoration with Restore Stripping buffer (Thermo) and incubated with rabbit monoclonal antibody GAPDH (CST-14C10) Cell Signaling Technologies. (d) Chemiluminescent acquisition using Bio-rad Chemidoc of the nitrocellulose membrane that contains a prestained ladder incubated with a streptavidin HRP incubated in a dilution of 1:10000 along with secondary antibody, and proteins derived from DAOY single cell colonies. Primary antibodies used was rabbit monoclonal antibody YAP1 (CST-D8H1X) Cell Signaling Technologies, rabbit monoclonal antibody GAPDH (CST-14C10) and Secondary HRP-Rabbit (7074S). Figure S2: (a) Dose curve comparison of DAOY and UW228 using Sonidegib normalized with DMSO. Control versus YAP1 Depleted cells (YAP1-KO). Conditions for functional assays detailed in the manuscript body was 18.2uM in 24 hours for DAOY and 15uM in 48h for UW228. (b) Correlation between *YAP1* and *GLI1* or *GLI2* using RNAseq data from 46 SHH MB samples. Analysis was performed on R2 genomic visualization platform (https://hgserver1.amc.nl/cgi-bin/r2/main.cgi, accessed on 29 November 2021). (c) t-SNE map generated by Seurat for single-cell data analysis of 5 SHH MB samples MUV41, SJ454, SJ577, RCMB18 and RCMB24. Gene Expression Omnibus (GSE119926) (Top left). Samples setting PDX or Diagnostic (Top right). Cellular populations (Center bottom). Table S1: Correlated pathways associated with *YAP1* expression in SHH MB samples of GSE85217 dataset. Output table from Algorithmic Annotation of Functional Roles for Components of 3044 Human Molecular Pathways [25].

## References

[1] Cavalli F.M., Remke M., Rampasek L., Peacock J., Shih D.J.H., Luu B., Garzia L., Torchia J., Nor C., Morrissy S. (2017). Intertumoral Heterogeneity within Medulloblastoma Subgroups. Cancer Cell.

[2] Northcott P.A., Robinson G.W., Kratz C.P., Mabbott D.J., Pomeroy S.L., Clifford S.C., Rutkowski S., Ellison D.W., Malkin D., Taylor M. (2019). Medulloblastoma. Nat. Rev. Dis. Prim..

[3] Ramaswamy V., Remke M., Bouffet E., Faria C.C., Perreault S., Cho Y.-J., Shih D.J., Luu B., Dubuc A.M., A Northcott P. (2013). Recurrence patterns across medulloblastoma subgroups: An integrated clinical and molecular analysis. Lancet Oncol..

[4] Kool M., Jones D.T.W., Jaeger N., Northcott P.A., Pugh T.J., Hovestadt V., Piro R.M., Esparza L.A., Markant S.L., Remke M. (2014). Genome Sequencing of SHH Medulloblastoma Predicts Genotype-Related Response to Smoothened Inhibition. Cancer Cell.

[5] Schwalbe E., Lindsey J.C., Nakjang S., Crosier S., Smith A.J., Hicks D., Rafiee G., Hill R.M., Iliasova A., Stone T. (2017). Novel molecular subgroups for clinical classification and outcome prediction in childhood medulloblastoma: A cohort study. Lancet Oncol..

[6] Cruzeiro G.A.V., Lira R., de Almeida Magalhães T., Scrideli C., Valera E.T., Baumgartner M., Tone L.G. (2020). CTGF expression is indicative of better survival rates in patients with medulloblastoma. Cancer Gene Ther..

[7] Robinson M.H., Maximov V., Lallani S., Farooq H., Taylor M.D., Read R.D., Kenney A.M. (2019). Upregulation of the chromatin remodeler HELLS is mediated by YAP1 in Sonic Hedgehog Medulloblastoma. Sci. Rep..

[8] Huang A., Garraway L.A., Ashworth A., Weber B. (2019). Synthetic lethality as an engine for cancer drug target discovery. Nat. Rev. Drug Discov..

[9] Cruzeiro G.A.V., Salomão K.B., de Biagi C.A.O., Baumgartner M., Sturm D., Lira R., Magalhães T.D.A., Milan M.B., Silveira V.D.S., Saggioro F.P. (2019). A simplified approach using Taqman low-density array for medulloblastoma subgrouping. Acta Neuropathol. Commun..

[10] Triscott J., Lee C., Foster C., Manoranjan B., Pambid M.R., Berns R., Fotovati A., Venugopal C., O’Halloran K., Narendran A. (2013). Personalizing the Treatment of Pediatric Medulloblastoma: Polo-like Kinase 1 as a Molecular Target in High-Risk Children. Cancer Res..

[11] Ivanov D.P., Coyle B., Walker D.A., Grabowska A.M. (2016). In vitro models of medulloblastoma: Choosing the right tool for the job. J. Biotechnol..

[12] Greenfield E.A. (2019). Single-Cell Cloning of Hybridoma Cells by Limiting Dilution. Cold Spring Harb. Protoc..

[13] De Almeida Magalhães T., Cruzeiro G.A.V., de Sousa G.R., Da Silva K.R., Lira R.C.P., Scrideli C., Tone L.G., Valera E.T., Borges K.S. (2019). Notch pathway in ependymoma RELA-fused subgroup: Upregulation and association with cancer stem cells markers expression. Cancer Gene Ther..

[14] Klinger P.H.D.S., Delsin L.E.A., Cruzeiro G.A.V., Andrade A.F., Lira R.C.P., De Andrade P.V., Das Chagas P.F., Queiroz R.G.D.P., Trevisan F.A., De Oliveira R.S. (2020). Arsenic Trioxide exerts cytotoxic and radiosensitizing effects in pediatric Medulloblastoma cell lines of SHH Subgroup. Sci. Rep..

[15] Kieran M.W., Chisholm J., Casanova M., Brandes A.A., Aerts I., Bouffet E., Bailey S., Leary S., Macdonald T.J., Mechinaud F. (2017). Phase I study of oral sonidegib (LDE225) in pediatric brain and solid tumors and a phase II study in children and adults with relapsed medulloblastoma. Neuro-Oncology.

[16] Butler A., Hoffman P., Smibert P., Papalexi E., Satija R. (2018). Integrating single-cell transcriptomic data across different conditions, technologies, and species. Nat. Biotechnol..

[17] Stuart T., Butler A., Hoffman P., Hafemeister C., Papalexi E., Mauck W.M., Hao Y., Stoeckius M., Smibert P., Satija R. (2019). Comprehensive Integration of Single-Cell Data. Cell.

[18] Hovestadt V., Smith K.S., Bihannic L., Filbin M.G., Shaw M.L., Baumgartner A., DeWitt J.C., Groves A., Mayr L., Weisman H.R. (2019). Resolving medulloblastoma cellular architecture by single-cell genomics. Nat. Cell Biol..

[19] Keren-Paz A., Emmanuel R., Samuels Y. (2015). YAP and the drug resistance highway. Nat. Genet..

[20] Fernandez-L A., Northcott P.A., Dalton J., Fraga C., Ellison D., Angers S., Taylor M.D., Kenney A.M. (2009). YAP1 is amplified and up-regulated in hedgehog-associated medulloblastomas and mediates Sonic hedgehog-driven neural precursor proliferation. Genes Dev..

[21] Chou T.C. (2010). Drug Combination Studies and Their Synergy Quantification Using the Chou-Talalay Method. Cancer Res..

[22] Wang C., Zhu X., Feng W., Yu Y., Jeong K., Guo W., Lu Y., Mills G.B. (2015). Verteporfin inhibits YAP function through up-regulating 14-3-3σ sequestering YAP in the cytoplasm. Am. J. Cancer Res..

[23] Zhang L., He X., Liu X., Zhang F., Huang L., Potter A.S., Xu L., Zhou W., Zheng T., Luo Z. (2019). Single-Cell Transcriptomics in Medulloblastoma Reveals Tumor-Initiating Progenitors and Oncogenic Cascades during Tumorigenesis and Relapse. Cancer Cell.

[24] Raj N., Bam R. (2019). Reciprocal Crosstalk between YAP1/Hippo Pathway and the p53 Family Proteins: Mechanisms and Outcomes in Cancer. Front. Cell Dev. Biol..

[25] Sorokin M., Borisov N., Kuzmin D., Gudkov A., Zolotovskaia M., Garazha A., Buzdin A. (2021). Algorithmic Annotation of Functional Roles for Components of 3,044 Human Molecular Pathways. Front. Genet..

