# Supplementary material for: YAP1 Is a Potential Predictive Molecular Biomarker for Response to SMO Inhibitor in Medulloblastoma Cells
Source: Cancers (Basel). 2021 Dec 13;13(24):6249. doi: 10.3390/cancers13246249 (PMC8699675; doi:10.3390/cancers13246249)
Supplement: Supplementary file 1 [file cancers-13-06249-s001.zip › cancers-1404910-supplementary/Supplementary Figure 1.pdf]

Supplementary information
Supplemental figure

Fig. S1a

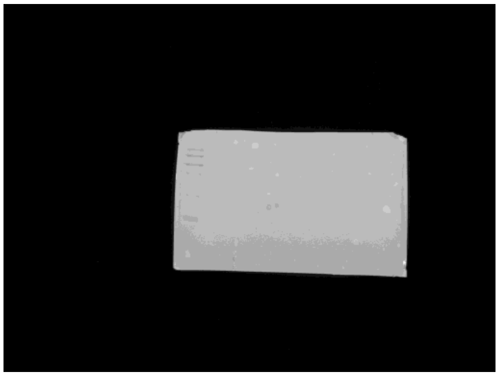

Fig. S1b

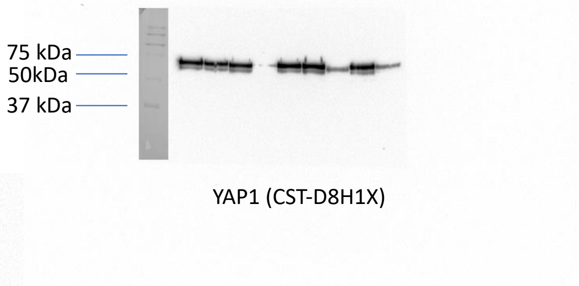

Fig. S1c

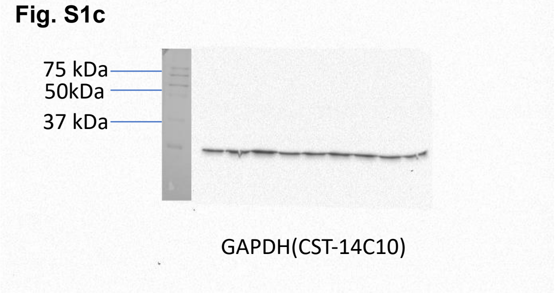

Fig. S1d

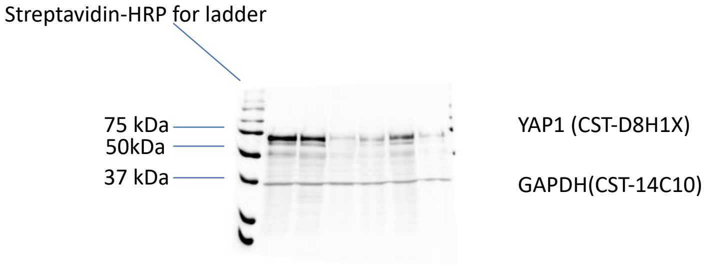

Fig.S1a. Colorimetric acquisition using Bio-rad Chemidoc of the nitrocellulose membrane that contains a prestained ladder and proteins derived from UW288 single cell colonies. **Fig.S1b** Chemiluminescent acquisition of nitrocellulose membrane from Fig2Sa incubated with rabbit monoclonal antibody YAP1 (CST-D8H1X) Cell Signaling Technologies and Secondary HRP-Rabbit (7074S). Fig. S3c Chemiluminescent acquisition of nitrocelulose membrane from **Fig.S1c** after restoration with Restore Stripping buffer (Thermo) and incubated with rabbit monoclonal antibody GAPDH (CST-14C10) Cell Signaling Technologies. **Fig.S1d.** Chemiluminescent acquisition using Bio-rad Chemidoc of the nitrocellulose

membrane that contains a prestained ladder incubated with a streptavidin HRP incubated in a dilution of 1:10000 along with secondary antibody, and proteins derived from DAOY single cell colonies. Primary antibodies used was rabbit monoclonal antibody YAP1 (CST-D8H1X) Cell Signaling Technologies, rabbit monoclonal antibody GAPDH (CST-14C10) and Secondary HRP-Rabbit (7074S).
