# Supplementary material for: YAP1 Is a Potential Predictive Molecular Biomarker for Response to SMO Inhibitor in Medulloblastoma Cells
Source: Cancers (Basel). 2021 Dec 13;13(24):6249. doi: 10.3390/cancers13246249 (PMC8699675; doi:10.3390/cancers13246249)
Supplement: Supplementary file 1 [file cancers-13-06249-s001.zip › cancers-1404910-supplementary/Supplementary Figure 2.pdf]

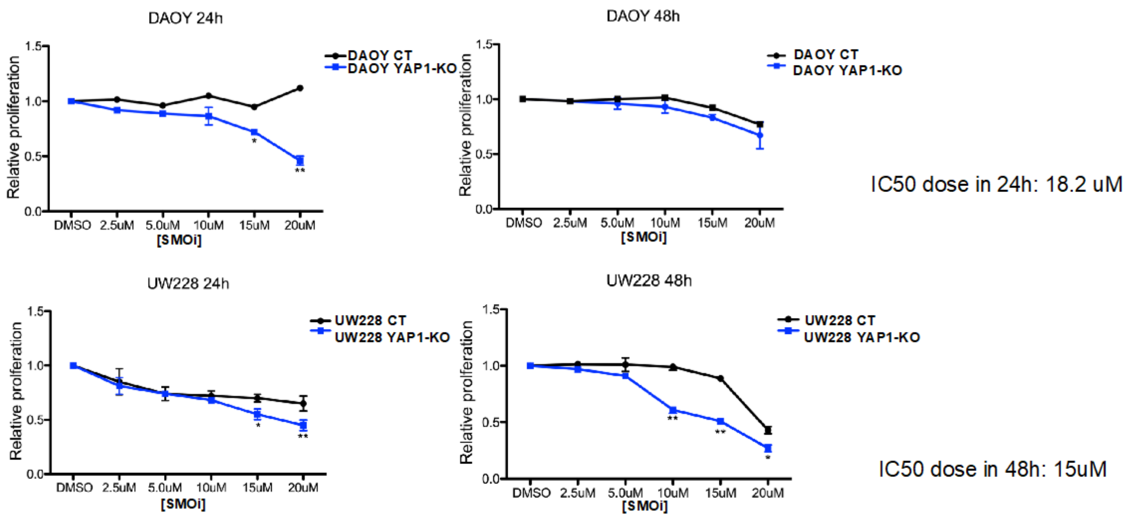
DAOY 24h
DAOY CT
DAOY YAP1-KO
Relative proliferation
DMSO 2.5uM 5.0uM 10uM 15uM 20uM
[SMOi]
DAOY 48h
DAOY CT
DAOY YAP1-KO
Relative proliferation
DMSO 2.5uM 5.0uM 10uM 15uM 20uM
[SMOi]
IC50 dose in 24h: 18.2 uM
UW228 24h
UW228 CT
UW228 YAP1-KO
Relative proliferation
DMSO 2.5uM 5.0uM 10uM 15uM 20uM
[SMOi]
UW228 48h
UW228 CT
UW228 YAP1-KO
Relative proliferation
DMSO 2.5uM 5.0uM 10uM 15uM 20uM
[SMOi]
IC50 dose in 48h: 15uM

(a)

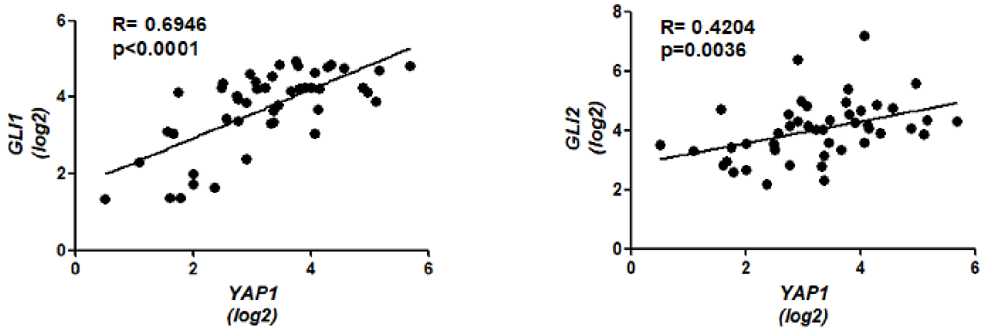
R= 0.6946
p<0.0001
GLI1 (log2)
YAP1 (log2)
R= 0.4204
p=0.0036
GLI2 (log2)
YAP1 (log2)

(b)

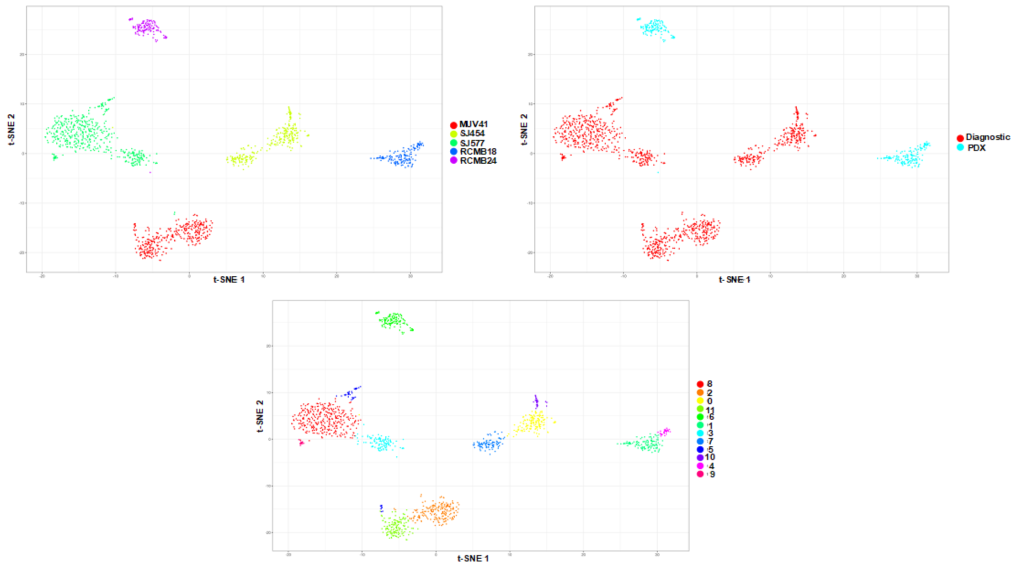
t-SNE 1
t-SNE 2
MJV41
SJ454
SJ577
RCMB18
RCMB24
Diagnostic
PDX

(c)

Fig.S2a Dose curve comparison of DAOY and UW228 using Sonidegib normalized with DMSO. Control versus YAP1 Depleted cells (YAP1-KO). Conditions for functional assays detailed in the manuscript body was 18.2uM in 24 hours for DAOY and 15uM in 48h for UW228. **Fig. S2b** Correlation between *YAP1* and *GLI1* or *GLI2* using RNAseq data from 46 SHH MB samples. Analysis was performed on R2 genomic visualization platform (https://hgserver1.amc.nl/cgi-bin/r2/main.cgi). **Fig. S2c** t-SNE map generated by Seurat for single-cell data analysis of 5 SHH MB samples MUV41, SJ454, SJ577, RCMB18 and RCMB24. Gene Expression Omnibus (GSE119926) (Top left). Samples setting PDX or Diagnostic (Top right). Cellular populations (Center bottom)
