# Supplementary material for: YAP1 Is a Potential Predictive Molecular Biomarker for Response to SMO Inhibitor in Medulloblastoma Cells
Source: Cancers (Basel). 2021 Dec 13;13(24):6249. doi: 10.3390/cancers13246249 (PMC8699675; doi:10.3390/cancers13246249)
Supplement: Supplementary file 1 [file cancers-13-06249-s001.zip › cancers-1404910-supplementary/Supplementary Figure 3.pdf]

Blots from figure 2A and 2B
Cropped Lanes

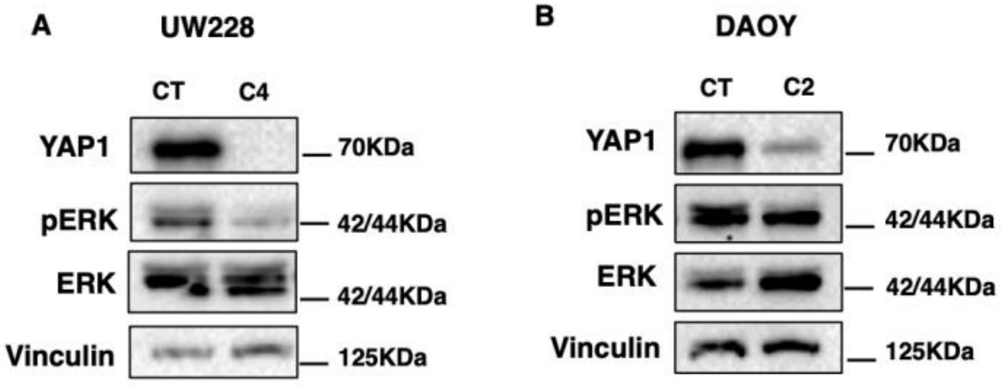

Uncropped Lanes

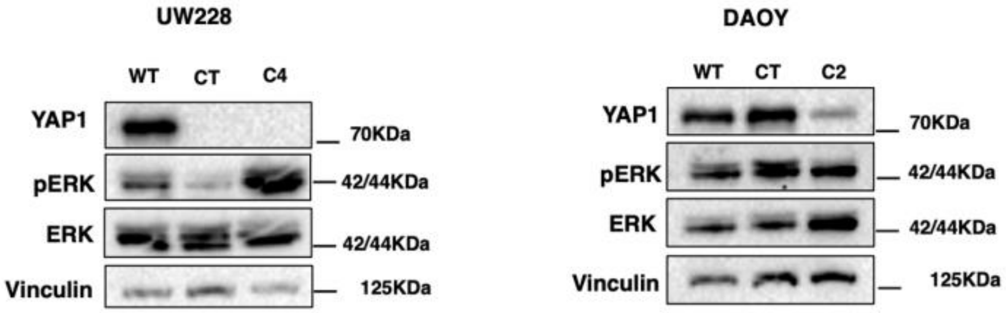

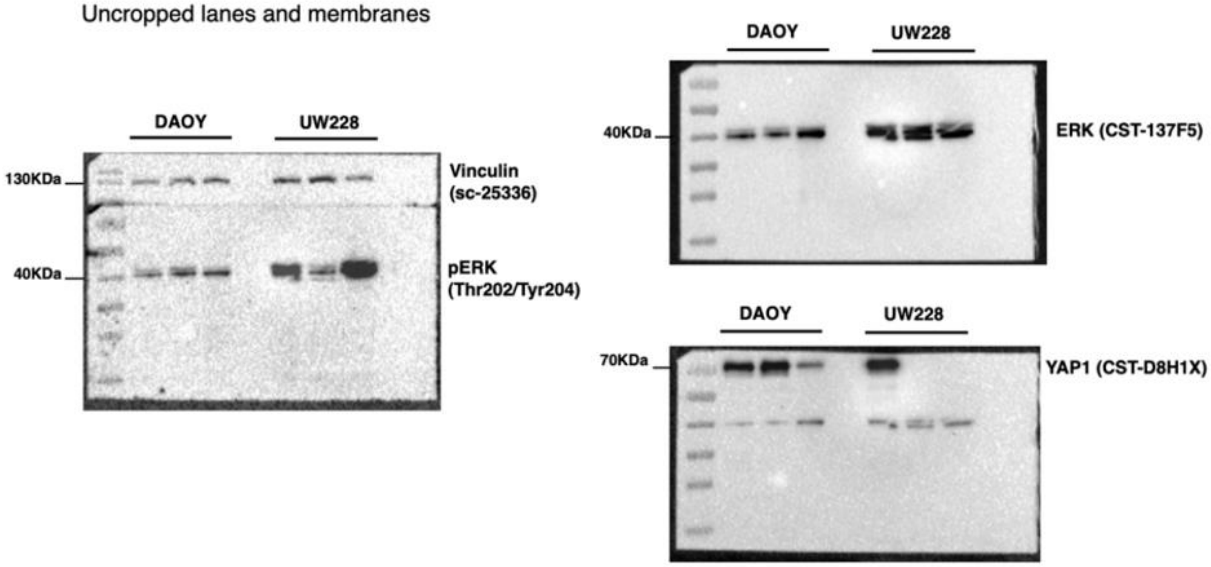

Figure S3. The uncropped lanes and membranes of Figure 2A and B.
